# Supplementary material for: Quantifying the photocurrent fluctuation in quantum materials by shot noise
Source: Nat Commun. 2024 Mar 5;15:2012. doi: 10.1038/s41467-024-46264-1 (PMC10914713; doi:10.1038/s41467-024-46264-1)
Supplement: Supplementary file 1 — Supplementary Information [file 41467_2024_46264_MOESM1_ESM.pdf]

# Supplementary Information
# to
# Quantifying the Photocurrent Fluctuation in Quantum Materials by Shot Noise

Longjun Xiang,[1] Hao Jin,[1] and Jian Wang[1,2,3,*]

[1]College of Physics and Optoelectronic Engineering, Shenzhen University, Shenzhen 518060, China
[2]Department of Physics, University of Hong Kong, Pokfulam Road, Hong Kong, China
[3]Department of Physics, The University of Science and Technology of China, Hefei, China

(Dated: February 20, 2024)

## CONTENTS

1. Supplementary Note 1: The second-quantization photocurrent operator 1
  1.1. The second-quantization density matrix operator 2
  1.2. The second-quantization photocurrent operator 3
  1.3. The shift and injection photocurrent 4

2. Supplementary Note 2: The shift and injection DSNs 5
  2.1. The double-time correlation function for the photocurrent operator 5
  2.2. The DC contribution from the double-time correlation function 6
  2.3. The shift DSN 8
  2.4. The injection DSN 9

3. Supplementary Note 3: The remaining nonvanishing DSNs 9

4. Supplementary Note 4: The influence of spin-orbit coupling 9

## 1. SUPPLEMENTARY NOTE 1: THE SECOND-QUANTIZATION PHOTOCURRENT OPERATOR

Following the noise theory formulated in mesoscopic conductors [1], the very first step to develop the noise theory for photocurrent is writing down the second-quantization photocurrent operator in terms of creation and annihilation operators, which encodes the electron occupation information or quantum statistical information. Recall that the photocurrent expectation value is calculated by [2, 3] ($e = \hbar = 1$)

$$J^a = \mathrm{Tr}[\hat{\rho}\hat{v}^a] = \sum_{nm} \int_k \rho_{mn} v^a_{nm}, \tag{S1}$$

where $\int_k \equiv \frac{1}{V} \int \frac{d\mathbf{k}}{(2\pi)^d}$ with $d$ the spatial dimensionality, $\hat{\rho}$ and $\hat{v}^a$ are the first-quantization density matrix operator and current operator, respectively. And the current at the $i$th order of optical electric field is obtained by iteratively solving the Liouville equation for $\rho^{(i)}_{nm}$ at the same order starting from [2, 3] $\rho^{(0)}_{mn} = \delta(0)\delta_{mn} f_m$, where $\delta(0) = \delta(\mathbf{k} - \mathbf{k})$ and $f_m$ is the equilibrium Fermi distribution function. Explicitly, one has

$$J^{a,(i)} = \sum_{nm} \int_k \rho^{(i)}_{mn} v^a_{nm}. \tag{S2}$$

Therefore, this method employed to calculate the photocurrent expectation value can not be directly extended to evaluate the photocurrent correlation function or to develop the noise theory for photocurrent. One one hand, the statistical information is encoded into density matrix element $\rho_{mn}$ instead of the first-quantization current operator $\hat{v}^a$ so that we can not appropriately consider the current correlation; on the other hand, the $\mathrm{Tr}[\cdots]$ used to calculate

* jianwang@hku.hk

the photocurrent expectation value usually considers both quantum average and quantum statistical average, while the evaluation of noise spectrum only takes the quantum statistical average into account.

Fortunately, by taking a close look at Eq. (S2), we find that the electron occupation information or statistical information is fully encoded into the density matrix element $\rho^{(i)}_{nm}$ and therefore, by "quantizing" the density matrix element $\rho^{(i)}_{nm}$ into second-quantization form, we can define a desirable second-quantization photocurrent operator, as explained in the main text. To be specific, we have

$$\hat{J}^{a,(i)} \equiv \sum_{nm} \int_k \hat{\rho}^{(i)}_{mn}(t) v^a_{nm} \equiv \sum_{nm} \int_k J^{a,(i)}_{mn}(t) a^\dagger_m a_n, \tag{S3}$$

as given by Eq. (2) in the main text, where $\hat{\rho}^{(i)}_{nm}(t)$ is the second-quantization density matrix element operator and $J^{a,(i)}_{nm}(t)$ the matrix element for the second-quantization photocurrent operator, as will be derived in subsections (1.1) and (1.2), respectively. Note that when "quantizing" $\rho^{(i)}_{mn}$, the quantum statistical average denoted by $\langle \cdots \rangle_s$ will naturally be introduced, which underlines that the current expectation value and current correlation function can be evaluated on the equal footing, the same as the noise theory formulated in mesoscopic conductors [1]. In addition, we remark that Eq. (S3) after taking the quantum statistical average recovers Eq. (S2) and therefore we have

$$\langle \hat{\rho}^{(i)}_{mn} \rangle_s = \rho^{(i)}_{mn}. \tag{S4}$$

### 1.1. The second-quantization density matrix operator

At the zeroth order of optical electric field, we find [2, 3]

$$\rho^{(0)}_{mn} = \delta(0)\delta_{nm} f_m = \langle a^\dagger_m a_n \rangle_s, \tag{S5}$$

where $\langle \cdots \rangle_s$ means quantum statistical average, and hence we immediately obtain

$$\hat{\rho}^{(0)}_{mn} = a^\dagger_m a_n, \tag{S6}$$

where $a^\dagger_m$ and $a_n$ are the creation and annihilation operators for the Bloch states, respectively. Note that both $a^\dagger_n$ and $a_m$ do not evolve as time. Furthermore, by requiring $\langle \hat{\rho}^{(i)}_{nm} \rangle_s = \rho^{(i)}_{nm}$, we find that $\hat{\rho}^{(i)}_{mn}$ with $i \geq 1$ can be obtained by iteratively solving the Liouville equation [2, 3]:

$$i\partial_t \hat{\rho}_{mn} = \omega_{mn} \hat{\rho}_{mn} + \left[ i D^b_{mn} \hat{\rho}_{mn} + \sum_l \left( r^b_{ml} \hat{\rho}_{ln} + \hat{\rho}_{ml} r^b_{ln} \right) \right] E^b(t), \tag{S7}$$

where $\omega_{mn} = \omega_m - \omega_n$, $D^b_{mn} \equiv \partial_b - i(\mathcal{A}^b_m - \mathcal{A}^b_n)$ the covariant derivative with $\partial_b \equiv \partial/\partial k_b$ and $\mathcal{A}^b_n$ the intraband Berry connection, $r^b_{ml}$ the interband Berry connection, and $E^b(t) = E^b_\beta e^{-i\omega_\beta t} + c.c.$ the monochromatic optical electric field, where $E^b_\beta \equiv E^b(\omega_\beta)$ and $c.c.$ the complex conjugate of the first term. Then by writing $\hat{\rho}_{mn} = \sum_{n=1}^{\infty} \hat{\rho}^{(n-1)}_{mn}$, where $\hat{\rho}^{(n-1)}_{mn}$ is proportional to $\left(E^b_\beta\right)^{(n-1)}$, Eq. (S7) becomes

$$i\partial_t \hat{\rho}^{(n)}_{mn} = \omega_{mn} \hat{\rho}^{(n)}_{mn} + \left( i D^b_{mn} \hat{\rho}^{(n-1)}_{mn} + \hat{q}^{b,(n-1)}_{mn} \right) E^b(t), \tag{S8}$$

where

$$\hat{q}^{b,(n-1)}_{mn} \equiv \sum_l \left( r^b_{ml} \hat{\rho}^{(n-1)}_{ln} - \hat{\rho}^{(n-1)}_{ml} r^b_{ln} \right). \tag{S9}$$

Substituting Eq. (S6) into Eq. (S8), we find

$$\hat{\rho}^{(1)}_{mn} = \frac{i D^b_{mn} \hat{\rho}^{(0)}_{mn} + \hat{q}^{b,(0)}_{mn}}{\omega_\beta - \omega_{mn}} E^b_\beta e^{-i\omega_\beta t} \equiv \hat{\rho}^{b\beta}_{mn} E^b_\beta e^{-i\omega_\beta t}, \tag{S10}$$

where $\omega_\beta$ contains an infinitesimal quantity $+i\eta$ with $\eta \to 0^+$ and the summation over the field indices, namely $b$ and $\beta$, are assumed.

Furthermore, by substituting Eq. (S10) into Eq. (S8), we find

$$\begin{aligned}\hat{\rho}^{(2)}_{mn} &= \frac{iD^c_{mn}\hat{\rho}^{b\beta}_{mn} + \sum_l (r^c_{ml}\hat{\rho}^{b\beta}_{ln} - \hat{\rho}^{b\beta}_{ml} r^c_{ln})}{\omega_\Sigma - \omega_{mn}} E^b_\beta E^c_\gamma e^{-i\omega_\Sigma t}, \\ &= \frac{1}{\omega_\Sigma - \omega_{mn}} \left[ -D^c_{mn} \left( \frac{D^b_{mn}\hat{\rho}^{(0)}_{mn}}{\omega_\beta - \omega_{mn}} \right) + iD^c_{mn} \left( \frac{\hat{q}^{b,(0)}_{mn}}{\omega_\beta - \omega_{mn}} \right) \right] E^b_\beta E^c_\gamma e^{-i\omega_\Sigma t} \\ &+ \frac{1}{\omega_\Sigma - \omega_{mn}} \sum_l \left[ r^c_{ml} \left( \frac{iD^b_{ln}\hat{\rho}^{(0)}_{ln} + \hat{q}^{b,(0)}_{ln}}{\omega_\beta - \omega_{ln}} \right) - \left( \frac{iD^b_{ml}\hat{\rho}^{(0)}_{ml} + \hat{q}^{b,(0)}_{ml}}{\omega_\beta - \omega_{ml}} \right) r^c_{ln} \right] E^b_\beta E^c_\gamma e^{-i\omega_\Sigma t}, \end{aligned} \tag{S11}$$

where $\omega_\Sigma = \omega_\beta + \omega_\gamma$. For $m \neq n$, we have:

$$\begin{aligned}\hat{\rho}^{(2)}_{mn} &= \frac{1}{-\omega_{mn}} \left[ -D^c_{mn} \left( \frac{D^b_{mn}\hat{\rho}^{(0)}_{mn}}{\omega_\beta - \omega_{mn}} \right) + iD^c_{mn} \left( \frac{\hat{q}^{b,(0)}_{mn}}{\omega_\beta - \omega_{mn}} \right) \right] E^b_\beta E^c_\gamma e^{-i\omega_\Sigma t} \\ &+ \frac{1}{-\omega_{mn}} \sum_l \left[ r^c_{ml} \left( \frac{iD^b_{ln}\hat{\rho}^{(0)}_{ln} + \hat{q}^{b,(0)}_{ln}}{\omega_\beta - \omega_{ln}} \right) - \left( \frac{iD^b_{ml}\hat{\rho}^{(0)}_{ml} + \hat{q}^{b,(0)}_{ml}}{\omega_\beta - \omega_{ml}} \right) r^c_{ln} \right] E^b_\beta E^c_\gamma e^{-i\omega_\Sigma t}. \end{aligned} \tag{S12}$$

For $m = n$, we find that $\hat{\rho}^{(2)}_{mn}$ becomes:

$$\begin{aligned}\hat{\rho}^{(2)}_{nn} &= \frac{1}{\omega_\Sigma \omega_\beta} \left[ -\partial_c \partial_b \hat{\rho}^{(0)}_{nn} + i\partial_c \hat{q}^{b,(0)}_{nn} \right] E^b_\beta E^c_\gamma e^{-i\omega_\Sigma t} \\ &+ \frac{1}{\omega_\Sigma} \sum_l \left[ r^c_{nl} \left( \frac{iD^b_{ln}\hat{\rho}^{(0)}_{ln} + \hat{q}^{b,(0)}_{ln}}{\omega_\beta - \omega_{ln}} \right) - \left( \frac{iD^b_{nl}\hat{\rho}^{(0)}_{nl} + \hat{q}^{b,(0)}_{nl}}{\omega_\beta - \omega_{nl}} \right) r^c_{ln} \right] E^b_\beta E^c_\gamma e^{-i\omega_\Sigma t}. \end{aligned} \tag{S13}$$

Eq. (S12) and Eq. (S13) will be employed to calculate the shift and injection photocurrent operator, respectively. Note that the lowest-order DC shot noise (DSN) can be obtained by considering the correlation between $\hat{J}^{a,(0)}$ and $\hat{J}^{a,(2)}$ and hence we stop to solve for $\hat{\rho}^{(i)}_{nm}$ with $i \geq 3$.

### 1.2. The second-quantization photocurrent operator

With the second-quantization density matrix element operator, we are ready to derive the matrix element for the second-quantization photocurrent operator by Eq. (S3). Particularly, at zeroth order of $E^b_\beta$, we obtain:

$$\hat{J}^{a,(0)} = \sum_{mn} \int_k v^a_{nm} a^\dagger_m a_n, \tag{S14}$$

from which we immediately find

$$J^{a,(0)}_{mn} = v^a_{nm}. \tag{S15}$$

At the first order of $E^b_\beta$, by substituting Eq. (S10) into Eq. (S3), the corresponding second-quantization photocurrent operator is given by:

$$\hat{J}^{a,(1)} = \sum_{mn} \int_k \left[ v^a_{nm} \frac{iD^b_{mn}\hat{\rho}^{(0)}_{mn}}{\omega_\beta - \omega_{mn}} + v^a_{nm} \sum_l \frac{r^b_{ml}\hat{\rho}^{(0)}_{ln} - \hat{\rho}^{(0)}_{ml} r^b_{ln}}{\omega_\beta - \omega_{mn}} \right] E^b_\beta e^{-i\omega_\beta t}, \tag{S16}$$

By comparing with the second term of Eq. (S3), we find

$$J^{a,(1)}_{mn}(t) = \left[ -iD^b_{nm} \left( \frac{v^a_{nm}}{\omega_\beta - \omega_{mn}} \right) + \sum_l \left( \frac{v^a_{nl} r^b_{lm}}{\omega_\beta - \omega_{ln}} - \frac{r^b_{nl} v^a_{lm}}{\omega_\beta - \omega_{ml}} \right) \right] E^b_\beta e^{-i\omega_\beta t}, \tag{S17}$$

where first term is obtained by integration by parts and the second term obtained by interchanging the dummy band indices.

Similarly, at the second order of $E^b_\beta$, by writing $\hat{J}^{a,(2)} \equiv \hat{J}^{a,(2)}_{\mathrm{O}} + \hat{J}^{a,(2)}_{\mathrm{D}}$, where $\hat{J}^{a,(2)}_{\mathrm{O}} = \sum^{n\neq m}_{nm} \int_k v^a_{nm}\hat{\rho}^{(2)}_{mn} = \sum_{nm}\int_k J^{a,(2)}_{\mathrm{O},mn} a^\dagger_m a_n$ and $\hat{J}^{a,(2)}_{\mathrm{D}} = \sum_n \int_k v^a_n \hat{\rho}^{(2)}_{nn} = \sum_{nm}\int_k J^{a,(2)}_{\mathrm{D},mn} a^\dagger_m a_n$ stand for the off-diagonal and diagonal contributions, respectively, we find

$$J^{a,(2)}_{\mathrm{O},mn}(t) = \left[-iD^b_{nm}\left(\frac{r^c_{nm;a}}{\omega_\beta - \omega_{mn}}\right) + \sum_l \left(\frac{r^c_{nl;a} r^b_{lm}}{\omega_\beta - \omega_{ln}} - \frac{r^b_{nl} r^c_{lm;a}}{\omega_\beta - \omega_{ml}}\right)\right] E^b_\beta E^c_\gamma e^{-i\omega_\Sigma t}, \tag{S18}$$

$$J^{a,(2)}_{\mathrm{D},mn}(t) = \frac{1}{\omega_\Sigma}\left[iD^b_{nm}\left(\frac{\Delta^a_{mn} r^c_{nm}}{\omega_\beta - \omega_{mn}}\right) + \sum_l \left(\frac{\Delta^a_{nl} r^c_{nl} r^b_{lm}}{\omega_\beta - \omega_{ln}} - \frac{\Delta^a_{lm} r^b_{nl} r^c_{lm}}{\omega_\beta - \omega_{ml}}\right)\right] E^b_\beta E^c_\gamma e^{-i\omega_\Sigma t}$$
$$+ \frac{1}{\omega_\Sigma \omega_\beta}\left[-\partial_c \partial_b v^a_n \delta_{nm} - i r^b_{nm} \partial_c \Delta^a_{nm}\right] E^b_\beta E^c_\gamma e^{-i\omega_\Sigma t}, \tag{S19}$$

where we have defined $r^b_{nm;a} \equiv D^a_{nm} r^b_{nm}$ and used the relations [3] $v^a_{nm}/\omega_{nm} = i r^a_{nm}$ and $r^b_{nm;a} - r^a_{nm;b} = i\sum_l (r^a_{nl} r^b_{lm} - r^b_{nl} r^a_{lm})$. Note that $\hat{\rho}^{(2)}_{mn}$ with $m \neq n$ and $\hat{\rho}^{(2)}_{nn}$ defined in Eq. (S12) and Eq. (S13), respectively, have been used. In addition, we remark that the quantum statistical average of the $\hat{J}^{a,(2)}_{\mathrm{O}}$ ($\hat{J}^{a,(2)}_{\mathrm{D}}$) gives the shift (injection) photocurrent [see subsection (1.3)], and hence $\hat{J}^{a,(2)}_{\mathrm{O}}$ ($\hat{J}^{a,(2)}_{\mathrm{D}}$) is defined as shift (injection) second-quantization photocurrent current operator, which also plays the essential role to obtain the shift (injection) DSN.

### 1.3. The shift and injection photocurrent

With Eq. (S18), we find that

$$J^{a,(2)}_{\mathrm{O}} \equiv \sum_{mn}\int_k J^{a,(2)}_{\mathrm{O},mn}(t)\langle a^\dagger_m a_n\rangle_s = \sum_{nm}\int \frac{d\mathbf{k}}{(2\pi)^d} f_{nm}\frac{r^c_{nm;a} r^b_{mn}}{\omega_\beta - \omega_{mn}} E^b_\beta E^c_\gamma e^{-\omega_\Sigma t}, \tag{S20}$$

where $f_{nm} = f_n - f_m$ and $\delta(0)/V \equiv 1$ has been used. Note that the first term in Eq. (S18) is zero by requiring $m = n$ while this term is important to the shift DSN, as will be shown below. Furthermore, by symmetrizing $b\beta$ and $c\gamma$ [3], we obtain

$$J^{a,(2)}_{\mathrm{O}} = \frac{1}{2}\sum_{nm}\int \frac{d\mathbf{k}}{(2\pi)^d} f_{nm}\left(\frac{r^c_{nm;a} r^b_{mn}}{\omega_\beta - \omega_{mn} + i\eta} + \frac{r^b_{nm;a} r^c_{mn}}{\omega_\gamma - \omega_{mn} + i\eta}\right) E^b_\beta E^c_\gamma e^{-i\omega_\Sigma t}, \tag{S21}$$

where we have shown explicitly the infinitesimal quantity $i\eta$. Using the Sokhotski-Plemelj formula[3], the resonant part of Eq. (S21) is given by,

$$J^{a,(2)}_{\mathrm{O}} = \frac{i\pi}{2}\sum_{nm}\int \frac{d\mathbf{k}}{(2\pi)^d} f_{mn}\left(r^c_{nm;a} r^b_{mn} - r^c_{nm} r^b_{mn;a}\right)\delta(\omega_\beta - \omega_{mn}) E^b(\omega_\beta) E^c(-\omega_\beta) \tag{S22}$$

where we have used $\omega_\gamma = -\omega_\beta$. By summing over $\omega_\beta$ ($\omega_\beta = \pm\omega$) and defining $J^{a,(2)}_{\mathrm{O}} \equiv 2\sigma^{abc}_{2\mathrm{L}}\mathrm{Re}[E^b_\omega E^{c*}_\omega] + 2\sigma^{abc}_{2\mathrm{C}}\mathrm{Im}[E^b_\omega E^{c*}_\omega]$, we recover the the normal [3] and magnetic [4] shift photocurrent susceptibility tensor, respectively,

$$\sigma^{abc}_{2\mathrm{L}} = \frac{i\pi}{4}\sum_{nm}\int \frac{d\mathbf{k}}{(2\pi)^d} f_{mn}\left(M^{abc}_{mn} + M^{abc}_{mn}\right)\delta(\omega - \omega_{mn}), \tag{S23}$$

$$\sigma^{abc}_{2\mathrm{C}} = \frac{\pi}{4}\sum_{nm}\int \frac{d\mathbf{k}}{(2\pi)^d} f_{mn}\left(\bar{M}^{abc}_{mn} - \bar{M}^{acb}_{mn}\right)\delta(\omega - \omega_{mn}), \tag{S24}$$

where $M^{abc}_{mn} \equiv r^b_{mn} r^c_{nm;a} - r^b_{nm} r^c_{mn;a}$ and $\bar{M}^{abc}_{mn} \equiv r^b_{mn} r^c_{nm;a} + r^b_{nm} r^c_{mn;a}$. Note that we have used the following relations:

$$E^b(\omega)E^c(-\omega) = E^b_\omega E^{c*}_\omega = \mathrm{Re}[E^b_\omega E^{c*}_\omega] + i\mathrm{Im}[E^b_\omega E^{c*}_\omega], \tag{S25}$$

$$E^b(-\omega)E^c(\omega) = E^{b*}_\omega E^c_\omega = \left(E^b_\omega E^{c*}_\omega\right)^* = \mathrm{Re}[E^b_\omega E^{c*}_\omega] - i\mathrm{Im}[E^b_\omega E^{c*}_\omega]. \tag{S26}$$

Similarly, with Eq. (S19), we find

$$J^{a,(2)}_{\mathrm{D}} \equiv \sum_{mn}\int_k J^{a,(2)}_{\mathrm{D},mn}(t)\langle a^\dagger_m a_n\rangle_s = \frac{1}{\omega_\Sigma}\sum_{nm}\int \frac{d\mathbf{k}}{(2\pi)^d}\frac{\Delta^a_{nm} f_{nm} r^c_{nm} r^b_{mn}}{\omega_\beta - \omega_{mn}} E^b_\beta E^c_\gamma e^{-i\omega_\Sigma t}, \tag{S27}$$

where $\Delta^a_{nm} = \partial_a\omega_n - \partial_a\omega_m$. Note that only the second term in Eq. (S19) survives by requiring $m = n$ while the first term in Eq. (S19) can also contribute to the injection DSN, as shown below. Furthermore, by symmetrizing $b\beta$ and $c\gamma$, we have

$$J^{a,(2)}_{\mathrm{D}} = \frac{1}{2\omega_\Sigma}\sum_{nm}\int\frac{d\mathbf{k}}{(2\pi)^d}\Delta^a_{nm}f_{nm}\left[\frac{r^c_{nm}r^b_{mn}}{\omega_\beta-\omega_{mn}+i\eta}+\frac{r^b_{nm}r^c_{mn}}{\omega_\gamma-\omega_{mn}+i\eta}\right]E^b_\beta E^c_\gamma e^{-i\omega_\Sigma t}, \tag{S28}$$

where we have explicitly show the infinitesimal quantity $i\eta$. Moreover, taking the time derivative and using the Sokhotski-Plemelj formula, the resonant part of Eq. (S28) is given by,

$$\partial_t J^{a,(2)}_{\mathrm{D}} = \pi\sum_{nm}\int_k \Delta^a_{mn}f_{nm}r^c_{nm}r^b_{mn}\delta(\omega_\beta-\omega_{mn})E^b(\omega_\beta)E^c(-\omega_\beta), \tag{S29}$$

where we have taken $\omega_\gamma = -\omega_\beta$. By summing over $\omega_\beta$ ($\omega_\beta = \pm\omega$) and defining $\partial_t J^{a,(2)}_{\mathrm{D}} \equiv 2\eta^{abc}_{2\mathrm{L}}\mathrm{Re}[E^b_\omega E^{c*}_\omega] + 2\eta^{abc}_{2\mathrm{C}}\mathrm{Im}[E^b_\omega E^{c*}_\omega]$, we recover the magnetic [4] and normal [3] injection photocurrent susceptibility tensors, respectively,

$$\eta^{abc}_{2\mathrm{L}} = \frac{\pi}{2}\sum_{nm}\int\frac{d\mathbf{k}}{(2\pi)^d}\Delta^a_{mn}f_{nm}g^{cb}_{nm}\delta(\omega-\omega_{mn}), \tag{S30}$$

$$\eta^{abc}_{2\mathrm{C}} = \frac{\pi}{2}\sum_{nm}\int\frac{d\mathbf{k}}{(2\pi)^d}\Delta^a_{mn}f_{nm}\Omega^{cb}_{nm}\delta(\omega-\omega_{mn}), \tag{S31}$$

where $\Omega^{cb}_{nm} \equiv i(r^c_{nm}r^b_{mn} - r^b_{nm}r^c_{mn})$ is the local Berry curvature and $g^{cb}_{nm} \equiv r^c_{nm}r^b_{mn} + r^b_{nm}r^c_{mn}$ is the local quantum metric.

## 2. SUPPLEMENTARY NOTE 2: THE SHIFT AND INJECTION DSNS

In the previous section, the second-quantization photocurrent operators up to the second order of $E^b_\beta$, namely, Eq. (S15), Eq. (S17), Eq. (S18), and Eq. (S19) have been derived. In this section, we further derive the DC shot noises (DSNs) by evaluating their correlation at second order of $E^b_\beta$. In the calculation, some simplifications will be adopted based on physical arguments. Firstly, we will focus on the optical excitation near band edge throughout this work and hence we will ignore the contribution explicitly related to three-band and four-band processes [5]. Secondly, we will drop the Fermi-surface contribution, namely, terms involving $f'_n$, because we are interested in semiconducting or insulating materials. Thirdly, we will neglect the resonant contribution from $\delta(\omega)$ since the frequency of light is never zero. In addition, all nonresonant contribution will be discarded at the moment because the nonresonant contribution has an opposite dependence on the polarization of light with the resonant one, as dictated by the Sokhotski-Plemelj formula. For instance, the resonant shift DSN is nonzero only for circularly polarized light (CPL) while non-resonant shift DSN is nonzero only for linearly polarized light (LPL). The contribution of nonresonant terms will be studied in future.

### 2.1. The double-time correlation function for the photocurrent operator

Following the noise theory formulated in mesoscopic physics, the double-time correlation function can be similarly defined as:

$$\begin{aligned} S^{ab}(t,t') &\equiv \frac{1}{2}\langle\Delta\hat{J}^a(t)\Delta\hat{J}^b(t') + \Delta\hat{J}^b(t')\Delta\hat{J}^a(t)\rangle_s \\ &= \frac{1}{2}\left(\langle\hat{J}^a(t)\hat{J}^b(t')\rangle_s - \langle\hat{J}^a(t)\rangle_s\langle\hat{J}^b(t')\rangle_s + \langle\hat{J}^b(t')\hat{J}^a(t)\rangle_s - \langle\hat{J}^b(t')\rangle_s\langle\hat{J}^a(t)\rangle_s\right), \end{aligned} \tag{S1}$$

where $\Delta\hat{J}^a(t) = \hat{J}^a(t) - \langle\hat{J}^a(t)\rangle_s$ and $\hat{J}^a(t)$ is the second-quantization current operator derived in subsection (1.2), namely

$$\hat{J}^a(t) = \sum_{nm}\int_k J^a_{nm}(t)\hat{a}^\dagger_n\hat{a}_m, \tag{S2}$$

where we have suppressed the superscripts that indicate the order of optical electric field. Straightforwardly, by substituting Eq. (S2) into Eq. (S1), we find:

$$\begin{aligned} S^{ab}(t,t') = &\frac{1}{2}\sum_{mn}\sum_{m'n'}\int_k\int_{k'} J^a_{nm}(t)J^b_{n'm'}(t')\left[\langle a^\dagger_n a_m a^\dagger_{n'} a_{m'}\rangle_s - \langle a^\dagger_n a_m\rangle_s \langle a^\dagger_{n'} a_{m'}\rangle_s\right] \\ &+ \frac{1}{2}\sum_{mn}\sum_{m'n'}\int_k\int_{k'} J^b_{n'm'}(t')J^a_{nm}(t)\left[\langle a^\dagger_{n'} a_{m'} a^\dagger_{n} a_{m}\rangle_s - \langle a^\dagger_{n'} a_{m'}\rangle_s \langle a^\dagger_{n} a_{m}\rangle_s\right], \end{aligned} \tag{S3}$$

where $a^\dagger_n = a^\dagger_n(\mathbf{k})$, $a_m = a_m(\mathbf{k})$, $a^\dagger_{n'} = a^\dagger_{n'}(\mathbf{k}')$, $a_{m'} = a_{m'}(\mathbf{k}')$, $J^a_{nm}(t) = J^a_{nm}(\mathbf{k};t)$, and $J^b_{n'm'}(t') = J^a_{n'm'}(\mathbf{k}';t')$. At this stage, using [1]

$$\langle a^\dagger_n a_m a^\dagger_{n'} a_{m'}\rangle_s - \langle a^\dagger_n a_m\rangle_s \langle a^\dagger_{n'} a_m\rangle_s = \delta(\mathbf{k}-\mathbf{k}')\delta(\mathbf{k}-\mathbf{k}')\delta_{mn'}\delta_{nm'} f_n(1-f_m), \tag{S4}$$

we obtain

$$\begin{aligned} S^{ab}(t,t') &= \frac{1}{2}\sum_{mn}\int_k J^a_{nm}(t)J^b_{mn}(t')\left[f_n(1-f_m)+f_m(1-f_n)\right] \\ &= \frac{1}{2}\sum_{mn}\int_k J^b_{mn}(t')J^a_{nm}(t)\left[f_m(1-f_m)+f_n(1-f_n)\right] + \frac{1}{2}\sum_{mn}\int_k J^b_{mn}(t')J^a_{nm}(t)f^2_{nm}, \end{aligned}$$

where the first term gives the thermal noise, which features the Fermi-surface property due to $f_n(1-f_n) = -k_B T\partial f_n/\partial\epsilon_n$ and vanishes for gapped systems that we are interested in, while the second term gives the shot noise with $f_{nm} = f_n - f_m$ and features the Fermi-sea property. Note that $\delta(0)/V \equiv 1$ and $f_n(1-f_m)+f_m(1-f_n) = f^2_{nm} + f_n(1-f_n) + f_m(1-f_m)$ have been used. Furthermore, focusing on the autocorrelation ($a=b$), we find

$$S^{a,(i+j)}(t,t') = \frac{1}{2}\sum_{nm}\int_k J^{a,(i)}_{nm}(t)J^{a,(j)}_{mn}(t')f^2_{nm}, \tag{S5}$$

where $S^a \equiv S^{aa}$ and the superscripts $(i)$, $(j)$, $(i+j)$ are used to indicate the order of optical electric field.

Note that $S^{a,(i+j)}(t,t')$ is a function of two independent time variables and generally stands for an AC SN. However, by adopting a Wigner transformation,

$$\begin{cases} t_0 = (t+t')/2, \\ t_1 = t-t'. \end{cases} \tag{S6}$$

we obtain a new correlation function $S^{a,(i+j)}(t_1,t_0)$, where $t_1$ and $t_0$ stand for the short and long time scales, respectively. Next, by taking the time average over $t_0$, we can pick up the DC component of $S^{a,(i+j)}(t_1,t_0)$ for $t_0$ since we are usually interested in the noise spectrum on a time scale long compared to $1/\omega$, where $\omega$ is the driving frequency of the optical electric field. To be specific, we have

$$S^{a,(i+j)}(t_1) = \frac{1}{T}\int_0^T dt_0 S^{a,(i+j)}(t,t')\Big|^{t=t_0+t_1/2}_{t'=t_0-t_1/2}, \tag{S7}$$

where $T \equiv 2\pi/\omega$. Moreover, by performing a Fourier transform for $S^{a,(i+j)}(t_1)$, we obtain the SN spectrum $S^{a,(i+j)}(\Omega_1)$, where $\Omega_1$ is the response frequency for $t_1$. Particularly, at the second order of $E^b_\beta$, we extract a DSN $S^{a,(2)}(\Omega_1) = \delta(\Omega_1)S^{a,(2)}$, where $S^{a,(2)}$ is the equal-time correlation function that contains the shift and injection DSNs contributed by the correlation between $\hat{J}^{a,(0)}$ and $\hat{J}^{a,(2)}$. We remark that the correlation function of $\hat{J}^{a,(1)}$ at different times also contributes a second-order SN but in AC regime for $t_1$, as shown below. Finally, we remark that the strategy to extract the DC component from a general double-time correlation function $S^{a,(i+j)}(t,t')$ is the same as that adopted in mesoscopic conductors [1].

### 2.2. The DC contribution from the double-time correlation function

Up to the second order of the optical electric field, the matrix element in Eq. (S2) can be expanded as

$$J^a_{mn}(t) = J^{a,(0)}_{mn} + J^{a,(1)}_{mn}(t) + J^{a,(2)}_{\mathrm{O},mn}(t) + J^{a,(2)}_{\mathrm{D},mn}(t), \tag{S8}$$

where $J_{mn}^{a,(0)} = v_{nm}^a$ is the zeroth order contribution which does not depend on time, $J_{mn}^{a,(1)}(t) \equiv J_{mn}^{a,b\beta} e^{-i\omega_\beta t}$, $J_{\mathrm{O},mn}^{a,(2)}(t) \equiv J_{\mathrm{O},mn}^{a,b\beta c\gamma} e^{-i\omega_\Sigma t}$, and $J_{\mathrm{D},mn}^{a,(2)}(t) \equiv J_{\mathrm{D},mn}^{a,b\beta c\gamma} e^{-i\omega_\Sigma t}$ are the matrix elements for the second-quantization photocurrent operator, where the explicit expressions for $J_{mn}^{a,b\beta}$, $J_{\mathrm{O},nm}^{a,b\beta c\gamma}$, $J_{\mathrm{D},nm}^{a,b\beta c\gamma}$ without time dependence can be found from Eq. (S17), Eq. (S18) and Eq. (S19), respectively.

From Eq. (S5), the second-order shot noise from the correlation of $J_{nm}^{a,(0)}$ and $J_{\mathrm{O},mn}^{a,(2)}$ (defined as shift shot noise $S_{\mathrm{sht}}^{a,(2)}(t,t')$) is calculated as:

$$\begin{aligned} S_{\mathrm{sht}}^{a,(2)}(t,t') &= S_{\mathrm{sht}}^{a,(0+2)}(t,t') + S_{\mathrm{sht}}^{a,(2+0)}(t,t') \\ &= \frac{1}{2}\sum_{nm}\int_k f_{nm}^2 v_{mn}^a J_{\mathrm{O},mn}^{a,b\beta c\gamma} e^{-i\omega_\Sigma t'} + \frac{1}{2}\sum_{nm}\int_k f_{nm}^2 J_{\mathrm{O},nm}^{a,b\beta c\gamma} v_{nm}^a e^{-i\omega_\Sigma t} \\ &= \frac{1}{2}\sum_{nm}\int_k f_{nm}^2 v_{mn}^a J_{\mathrm{O},mn}^{a,b\beta c\gamma}\left(e^{-i\omega_\Sigma t} + e^{-i\omega_\Sigma t'}\right). \end{aligned} \tag{S9}$$

By solving Eq. (S6), we have

$$\begin{cases} t = t_0 + t_1/2, \\ t' = t_0 - t_1/2, \end{cases} \tag{S10}$$

and hence we find

$$S_{\mathrm{sht}}^{a,(2)}(t_1,t_0) = \sum_{nm}\int_k f_{nm}^2 v_{mn}^a J_{\mathrm{O},mn}^{a,b\beta c\gamma} e^{-i\omega_\Sigma t_0}\cos(\omega_\Sigma t_1/2). \tag{S11}$$

Taking average over $t_0$ in a period of $T = 2\pi/\omega$, we find $\omega_\Sigma = 0$ or $\omega_\beta = -\omega_\gamma$ (This condition in fact gives the DC shift photocurrent by taking $\langle \hat{J}_{\mathrm{O}}^{a,(2)}\rangle_s$) and

$$S_{\mathrm{sht}}^{a,(2)}(t_1) = \sum_{nm}\int_k f_{nm}^2 v_{mn}^a J_{\mathrm{O},mn}^{a,b\beta c\gamma}, \tag{S12}$$

which is independent of $t_1$. As a result, by performing a Fourier transform for the short time scale or time difference, we arrive at:

$$S_{\mathrm{sht}}^{a,(2)}(\Omega_1) = \delta(\Omega_1)\sum_{nm}\int_k f_{nm}^2 v_{mn}^a J_{\mathrm{O},mn}^{a,b\beta c\gamma} \equiv \delta(\Omega_1) S_{\mathrm{sht}}^{a,(2)} \tag{S13}$$

where

$$S_{\mathrm{sht}}^{a,(2)} \equiv \sum_{nm}\int_k f_{nm}^2 v_{mn}^a J_{\mathrm{O},mn}^{a,b\beta c\gamma} \tag{S14}$$

is the equal-time correlation between $J_{nm}^{a,(0)}$ and $J_{\mathrm{O},mn}^{a,(2)}$. Following a similar way, the injection shot noise is calculated as:

$$\partial_{t_0} S_{\mathrm{inj}}^{a,(2)}(\Omega_1) = \delta(\Omega_1)\sum_{nm}\int_k f_{nm}^2 v_{mn}^a J_{\mathrm{D},mn}^{a,b\beta c\gamma} \equiv \delta(\Omega_1) S_{\mathrm{inj}}^{a,(2)} \tag{S15}$$

where the time derivative for $t_0$ is due to the $1/\omega_\Sigma$ factor in $J_{\mathrm{D},mn}^{a,(2)}(t)$ and

$$\partial_{t_0} S_{\mathrm{inj}}^{a,(2)} \equiv \sum_{nm}\int_k f_{nm}^2 v_{mn}^a\left(-i\omega_\Sigma J_{\mathrm{D},mn}^{a,b\beta c\gamma}\right). \tag{S16}$$

In addition, the shot noise due to $J_{nm}^{a,(1)}(t)$ at different times is found to be

$$S^{a,(2)}(t,t') = \frac{1}{2}\sum_{nm}\int_k f_{nm}^2 J_{nm}^{a,b\beta} J_{mn}^{a,c\gamma}[e^{-i(\omega_\beta t+\omega_\gamma t')} + e^{-i(\omega_\beta t'+\omega_\gamma t)}]. \tag{S17}$$

Using Eq. (S10), we find

$$S^{a,(2)}(t_1,t_0)=\sum_{nm} f_{nm}^2 J_{nm}^{a,b\beta} J_{mn}^{a,c\gamma} e^{-i\omega_\Sigma t_0}\cos[(\omega_\beta-\omega_\gamma)t_1/2]. \tag{S18}$$

Taking the averaging over $t_0$, we also have $\omega_\Sigma=0$ or $\omega_\beta=-\omega_\gamma$ and find

$$S^{a,(2)}(t_1)=\sum_{nm}\int_k f_{nm}^2 J_{nm}^{a,b\beta} J_{mn}^{a,c\gamma}\cos[\omega_\beta t_1], \tag{S19}$$

which explicitly shows a time dependence on $t_1$ and hence belongs to the AC shot noise for short time scale. Taking the Fourier transform in $t_1$ time space, we arrive at

$$S^{a,(2)}(\Omega_1)=\frac{1}{2}\sum_{nm}\int_k f_{nm}^2 J_{nm}^{a,b\beta} J_{mn}^{a,c\gamma}\left[\delta(\Omega_1+\omega_\beta)+\delta(\Omega_1-\omega_\beta)\right]=\delta(\Omega_1-\omega)S^{a,(2)} \tag{S20}$$

where $\omega_\beta=\pm\omega$ has been used and $\delta(\Omega_1+\omega)$ is dropped due to $\Omega_1>0$ and $\omega>0$. In addition,

$$S^{a,(2)}\equiv\sum_{nm}\int_k f_{nm}^2 J_{nm}^{a,b\beta} J_{mn}^{a,c\gamma} \tag{S21}$$

is the equal-time autocorrelation from $J_{nm}^{a,(1)}$.

### 2.3. The shift DSN

In this subsection, we derive the shift DSN. In terms of Eq. (S18) and Eq. (S14), we find:

$$\begin{aligned}
S_{\rm sht}^a &= \sum_{nm}\int_k v_{mn}^a\left[-iD_{nm}^b\left(\frac{r_{nm;a}^c}{\omega_\beta-\omega_{mn}}\right)+\sum_l\left(\frac{r_{nl;a}^c r_{lm}^b}{\omega_\beta-\omega_{ln}}-\frac{r_{nl}^b r_{lm;a}^c}{\omega_\beta-\omega_{ml}}\right)\right] f_{nm}^2 E_\beta^b E_\gamma^c\\
&=\sum_{nm}\int_k\left[\frac{iv_{mn;b}^a r_{nm;a}^c}{\omega_\beta-\omega_{mn}}+\sum_l\left(\frac{r_{nl;a}^c r_{lm}^b}{\omega_\beta-\omega_{ln}}-\frac{r_{nl}^b r_{lm;a}^c}{\omega_\beta-\omega_{ml}}\right)v_{mn}^a\right] f_{nm}^2 E_\beta^b E_\gamma^c\\
&=\sum_{nm}\int_k\left[\frac{iv_{mn;b}^a r_{nm;a}^c}{\omega_\beta-\omega_{mn}}f_{nm}^2+\sum_l\frac{\left(r_{ml}^b v_{ln}^a f_{nl}^2-v_{ml}^a r_{ln}^b f_{lm}^2\right) r_{nm;a}^c}{\omega_\beta-\omega_{mn}}\right]E_\beta^b E_\gamma^c,
\end{aligned} \tag{S22}$$

where the first term with $v_{mn;b}^a\equiv D_{mn}^b v_{mn}^a$ is obtained by integration by parts. Below we will focus on the first term and ignore the second term because this term explicitly involves three-band process. For the first term, by symmetrizing $b\beta$ and $c\gamma$, we have:

$$S_{\rm sht}^a=\frac{i}{4}\sum_{nm}\int_k f_{nm}^2\left(\frac{v_{mn;b}^a r_{nm;a}^c}{\omega_\beta-\omega_{mn}}+\frac{v_{nm;c}^a r_{mn;a}^b}{\omega_\gamma-\omega_{mn}}\right)E_\beta^b E_\gamma^c, \tag{S23}$$

Using the Sokhotski-Plemelj formula, the resonant part of Eq. (S23) is given by,

$$S_{\rm sht}^a=\frac{\pi}{2}\sum_{nm}\int_k f_{nm}^2\left(v_{mn;b}^a r_{nm;a}^c+v_{nm;c}^a r_{mn;a}^b\right)\delta(\omega_\beta-\omega_{mn})E^b(\omega_\beta)E^c(-\omega_\beta), \tag{S24}$$

where we have used $\omega_\gamma=-\omega_\beta$. Furthermore, by summing over $\omega_\beta(\omega_\beta=\pm\omega)$ and defining $S_{\rm sht}^a=2\sigma_{\rm L}^{abc}{\rm Re}[E_\omega^b E_\omega^{c*}]+2\sigma_{\rm C}^{abc}{\rm Im}[E_\omega^b E_\omega^{c*}]$, we find

$$\sigma_{\rm L}^{abc}=\frac{\pi}{4}\sum_{nm}\int_k f_{nm}^2\left(v_{mn;b}^a r_{nm;a}^c+v_{nm;b}^a r_{mn;a}^c+v_{nm;c}^a r_{mn;a}^b+v_{mn;c}^a r_{nm;a}^b\right)\delta(\omega-\omega_{mn}), \tag{S25}$$

$$\sigma_{\rm C}^{abc}=\frac{i\pi}{4}\sum_{nm}\int_k f_{nm}^2\left(v_{mn;b}^a r_{nm;a}^c-v_{nm;b}^a r_{mn;a}^c+v_{nm;c}^a r_{mn;a}^b-v_{mn;c}^a r_{nm;a}^b\right)\delta(\omega-\omega_{mn}), \tag{S26}$$

as given in the main text (only the $\mathcal{T}$-even component).

### 2.4. The injection DSN

In this subsection, we derive the injection DSN. In terms of Eq. (S19) and Eq. (S16), we find:

$$\partial_{t_0} S^a_{\rm inj} = \sum_{nm}\int_k v^a_{mn}\left[D^b_{nm}\left(\frac{\Delta^a_{mn}r^c_{nm}}{\omega_\beta-\omega_{mn}}\right)-\sum_l i\left(\frac{\Delta^a_{nl}r^c_{nl}r^b_{lm}}{\omega_\beta-\omega_{ln}}-\frac{\Delta^a_{lm}r^b_{nl}r^c_{lm}}{\omega_\beta-\omega_{ml}}\right)\right]f^2_{nm}E^b_\beta E^c_\gamma$$
$$=\sum_{nm}\int_k\left[\frac{\Delta^a_{nm}v^a_{mn;b}r^c_{nm}}{\omega_\beta-\omega_{mn}}f^2_{nm}-\sum_l\frac{i\Delta^a_{nm}r^c_{nm}\left(v^a_{nl}r^b_{ml}f^2_{nl}-v^a_{lm}r^b_{ln}f^2_{lm}\right)}{\omega_\beta-\omega_{mn}}\right]E^b_\beta E^c_\gamma, \tag{S27}$$

where we have taken $\omega_\Sigma = 0$. Note that the second term also explicitly involves three-band process and we will only consider the first term below. For the first term, by symmetrizing $b\beta$ and $c\gamma$, we have:

$$\partial_{t_0} S^a_{\rm inj} = \frac{1}{2}\sum_{nm}\int_k\left(\frac{v^a_{mn;b}r^c_{nm}}{\omega_\beta-\omega_{mn}}+\frac{v^a_{mn;c}r^b_{nm}}{\omega_\gamma-\omega_{mn}}\right)\Delta^a_{nm}f^2_{nm}E^b_\beta E^c_\gamma. \tag{S28}$$

Furthermore, using the Sokhotski-Plemelj formula, the resonant part of Eq. (S28) is given by,

$$\partial_{t_0} S^a_{\rm inj} = -\frac{i\pi}{2}\sum_{nm}\int_k f^2_{nm}\Delta^a_{nm}\left(v^a_{mn;b}r^c_{nm}-v^a_{nm;c}r^b_{mn}\right)\delta(\omega_\beta-\omega_{mn})E^b(\omega_\beta)E^c(-\omega_\beta), \tag{S29}$$

where we have taken $\omega_\gamma = -\omega_\beta$. Furthermore, by summing over $\omega_\beta(\omega_\beta=\pm\omega)$ and defining $\partial_{t_0} S^a_{\rm inj} \equiv 2\eta^{abc}_{\rm L}{\rm Re}[E^b_\omega E^{c*}_\omega]+2\eta^{abc}_{\rm C}{\rm Im}[E^b_\omega E^{c*}_\omega]$, we find

$$\eta^{abc}_{\rm L} = +\frac{i\pi}{4}\sum_{nm}\int_k f^2_{nm}\Delta^a_{nm}\left(v^a_{mn;b}r^c_{nm}-v^a_{nm;c}r^b_{mn}-v^a_{nm;b}r^c_{mn}+v^a_{mn;c}r^b_{nm}\right)\delta(\omega-\omega_{mn}), \tag{S30}$$
$$\eta^{abc}_{\rm C} = -\frac{\pi}{4}\sum_{nm}\int_k f^2_{nm}\Delta^a_{nm}\left(v^a_{mn;b}r^c_{nm}-v^a_{nm;c}r^b_{mn}+v^a_{nm;b}r^c_{mn}-v^a_{mn;c}r^b_{nm}\right)\delta(\omega-\omega_{mn}). \tag{S31}$$

as given in the main text (only the $\mathcal{T}$-even component).

## 3. SUPPLEMENTARY NOTE 3: THE REMAINING NONVANISHING DSNS

For monolayer GeS with point groups (PGs) $mm2$ and $mmm$, we find that the nonvanishing injection DSN susceptibility tensors are $\eta^{xxx}_L$, $\eta^{xyy}_L$, $\eta^{xzz}_L$, $\eta^{yxx}_L$, $\eta^{yyy}_L$, and $\eta^{yzz}_L$, particularly by using the Bilbao database [6] with the Jahn notation $VV[V^2]$. Note that $\eta^{zxx}_L$, $\eta^{zyy}_L$, and $\eta^{zzz}_L$ with the photocurrent along the out-of-plane direction are zero in two-dimensional (2D) limit. In the main text, we only show $\eta^{xyy}_L$. The remaining components are shown in Supplementary Fig. 1 by defining $s^\eta = t_0\eta^{abc}_L E^2$.

Similarly, for bilayer $MoS_2$ with PGs $3R$ and $2H$, we find that the nonvanishing shift DSN susceptibility tensors are $\sigma^{yxz}_C = -\sigma^{yzx}_C = \sigma^{xzx}_C = -\sigma^{xxz}_C$, which contains only one independent component, as shown in the main text. In addition, the nonvanishing injection DSN susceptibility tensors are $\eta^{yxz}_{\rm L} = \eta^{yzx}_{\rm L} = -\eta^{xzx}_{\rm L} = -\eta^{xxz}_{\rm L}$, $\eta^{yyy}_{\rm L} = \eta^{xxx}_{\rm L}$, $\eta^{yxx}_{\rm L} = \eta^{xyy}_{\rm L}$, $\eta^{yzz}_{\rm L} = \eta^{xzz}_{\rm L}$, $\eta^{zyy}_{\rm L} = \eta^{zxx}_{\rm L}$, and $\eta^{zzz}_{\rm L}$. Note that $\eta^{zyy}_{\rm L} = \eta^{zxx}_{\rm L} = 0$ and $\eta^{zzz}_{\rm L} = 0$ in 2D limit and therefore the contributed independent components are $\eta^{xxx}_{\rm L}$, $\eta^{xyy}_{\rm L}$ and $\eta^{xzz}_{\rm L}$. In the main text, we only show $\eta^{xxx}_L$. The remaining components are shown in Supplementary Fig. 2 by defining $s^\eta = t_0\eta^{abc}_L E^2$.

## 4. SUPPLEMENTARY NOTE 4: THE INFLUENCE OF SPIN-ORBIT COUPLING

Note that in the main text, we have ignored the influence of spin-orbit coupling (SOC), which is believed to be important particularly in the case of $MoS_2$. In Supplementary Fig. 3(a) and Supplementary Fig. 3(b), we plot the shift and injection DSNs with the consideration of SOC, respectively. And we find that the influence of SOC for both DSNs is minor.

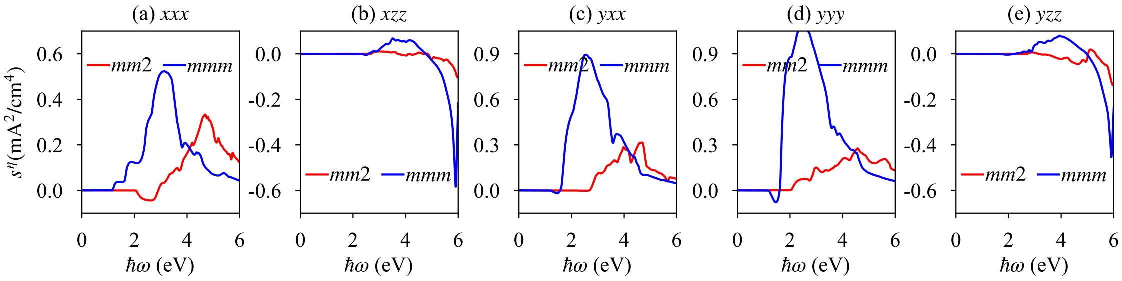


Supplementary Fig. 1: The remaining DC shot noise (DSN) for monolayer GeS. (a-e) The nonvansishing injection DSNs for monolayer GeS with point group $mm2$ and $mmm$, respectively.

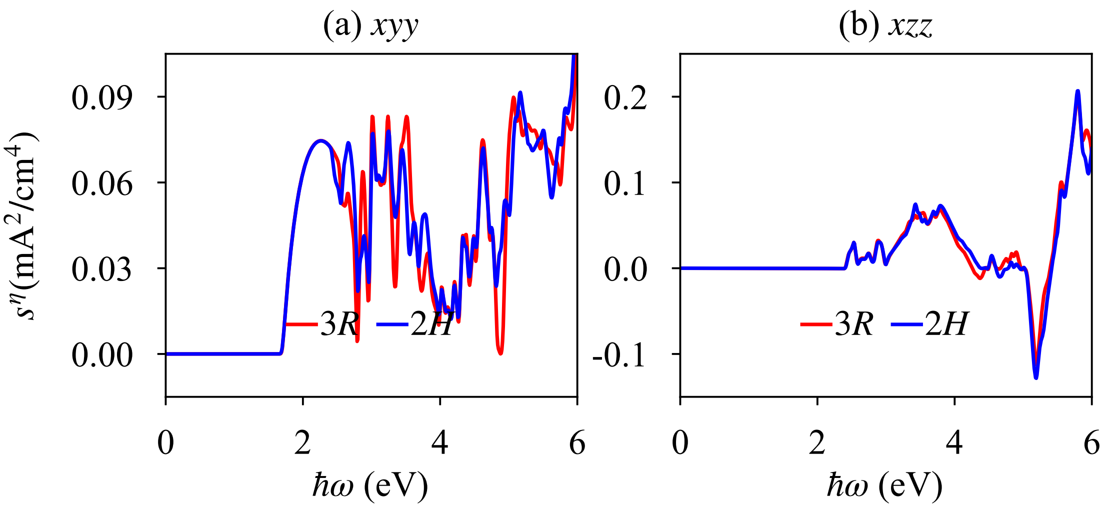


Supplementary Fig. 2: The remaining DC shot noise (DSN) for bilayer $MoS_2$. (a-b) The nonvansishing injection DSNs for bilayer $MoS_2$ with point group $3R$ and $2H$, respectively.

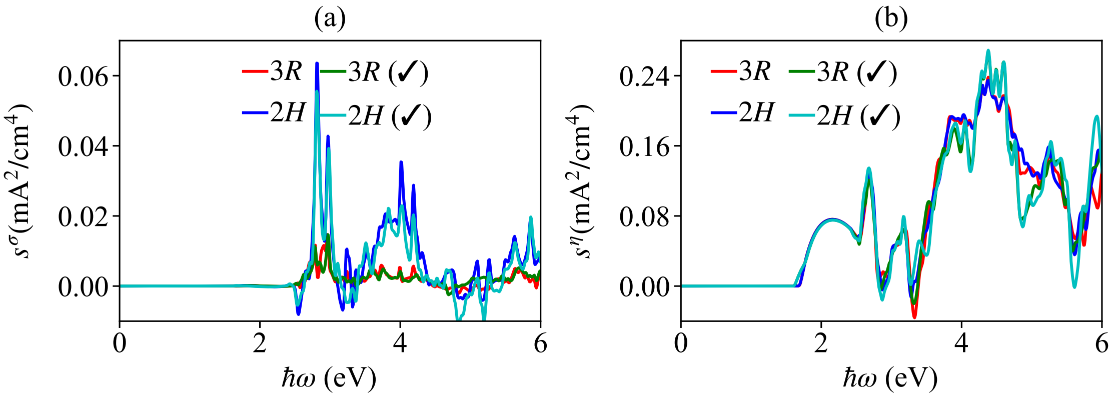


Supplementary Fig. 3: Influence of spin-orbit coupling (SOC) on DC shot noise (DSN) in $MoS_2$. The (a) shift and (b) injection DSNs for $MoS_2$ without and with the consideration of SOC. Here ✓ indicates that the SOC is considered.

## Supplementary References


[1] Blanter, Ya.M. & Büttiker, M. Shot noise in mesoscopic conductors. Phys. Rep. **336**, 1 (2000).

[2] Aversa, C. & Sipe, J. E. Nonlinear optical susceptibilities of semiconductors: Results with a length-gauge analysis. Phys.

Rev. B **52**, 14636 (1995).
[3] Sipe, J. E. & Shkrebtii, A. I. Second-order optical response in semiconductors. Phys. Rev. B **61**, 5337 (2000).
[4] Wang, H. & Qian, X.F. Electrically and magnetically switchable nonlinear photocurrent in $PT$-symmetric magnetic topological quantum materials. npj Comput. Mater. **6**, 199 (2020).
[5] Zhang, Y. et al. Switchable magnetic bulk photovoltaic effect in the two-dimensional magnet $CrI_3$. Nat. Commun. **10**, 3783 (2019).
[6] Gallego, S. V., Etxebarria, J., Elcoro, L., Tasci, E. S. & Perez-Mato, J. M. Automatic calculation of symmetry-adapted tensors in magnetic and non-magnetic materials: a new tool of the Bilbao Crystallographic Server. Acta Crystallogr. Sect. A**75**, 438 (2019).
